# Personalized Tests in Paralympic Athletes: Aerobic and Anaerobic Performance Profile of Elite Wheelchair Rugby Players

**DOI:** 10.3390/jpm10030118

**Published:** 2020-09-09

**Authors:** Giuseppe Marcolin, Nicola Petrone, Michael Benazzato, Francesco Bettella, Angela Gottardi, Luigi Salmaso, Livio Corain, Alfredo Musumeci, Stefano Masiero, Antonio Paoli

**Affiliations:** 1 Department of Biomedical Sciences, University of Padua, 35131 Padua, Italy; giuseppe.marcolin@unipd.it (G.M.); angigotti@gmail.com (A.G.); 2 Department of Industrial Engineering, University of Padua, 35131 Padua, Italy; nicola.petrone@unipd.it (N.P.); michael.benazzato@gmail.com (M.B.); fra.bettella@gmail.com (F.B.); 3 Department and Management and Engineering, University of Padua, 36100 Vicenza, Italy; luigi.salmaso@unipd.it (L.S.); livio.corain@unipd.it (L.C.); 4 Physical Medicine and Rehabilitation Unit, Department of Neuroscience, University of Padua, 35128 Padua, Italy; alfredomusumeci@yahoo.com (A.M.); stef.masiero@unipd.it (S.M.)

**Keywords:** invasion games, wheelchair sport, classification, Wingate test, sprint test, oxygen consumption

## Abstract

In Paralympic sports, the goal of functional classifications is to minimize the impact of impairment on the outcome of the competition. The present cross-sectional study aimed to investigate aerobic and anaerobic personalized tests in Paralympic athletes and to correlate them with the classification of the international wheelchair rugby federation (IWRF). Sixteen elite players of the Italian wheelchair rugby team volunteered for the study. Aerobic (incremental test to exhaustion) and anaerobic (Wingate 30s all-out test, 5 and 10-meter sprint test, shuttle test, isometric test) sport-performance measurements were correlated singularly or grouped (Z scores) with the classification point. Moreover, a multivariate permutation-based ranking analysis investigated possible differences in the overall level of performance among the adjacent classified groups of players, considering the scores of each test. A statistically significant correlation between the performance parameters and the IWRF functional classification considering both aerobic and anaerobic personalized tests was detected (0.58 ≤ r ≤ 0.88; 0.0260 ≤ *p* ≤ 0.0001). The multivariate permutation-based ranking analysis showed differences only for the low-pointers versus mid-pointers (*p* = 0.0195) and high-pointers (*p* = 0.0075). Although single performance parameters correlated with athletes’ classification point, results of the multivariate permutation-based ranking analysis seem to suggest considering only the most significant anaerobic and sport-specific performance parameters among athletes. These should be combined with the physical assessment and the qualitative observation, which are already part of the classification process to improve its effectiveness.

## 1. Introduction

Wheelchair rugby is a Paralympic team sport played on an indoor court, measuring 15 m in width and 28 m in length, where contacts among wheelchairs are part of the game [1]. In fact, athletes use their chairs to hold and block their opponents. Official matches are played in four periods of 8 min, and in the case of overtime, it lasts 3 min. The structure of the match determines the physical demand typical of this sport, which includes a repetition of short and intense exercises during the entire match [2] superimposed on a background of aerobic activity [3]. To be eligible to play wheelchair rugby, each athlete must have a disability that affects arms and legs. Most athletes present spinal lesions with full or partial paralysis, but other neurological health disorders are included among players (e.g., cerebral palsy, polio, muscular dystrophy, etc.) [1].

To overcome these impairments and to ensure fair and equiTable competitions, the International Wheelchair Rugby Federation (IWRF) introduced a classification system that ranks players in seven groups depending on their level of disability [4]. The point of each athlete ranks from 0.5 to 3.5, and the sum of the classification points of the four players on the court must be 8 or less [4]. The classification is based on three components: (i) Bench test, composed of manual muscle tests, trunk tests, and hand tests in all motion planes and a variety of situations; (ii) technical assessment, where sport and non-sport specific tests simulating wheelchair and ball activities are performed in a non-competitive environment, both with and without equipment; (iii) observation assessment, that is watching the player during training, warm-up, and competition [4]. It also includes the observation during time-outs, while the athlete is working with equipment, such as tape, gloves, water bottle straps, and binders [4]. The goal of the IWRF classification is to minimize the impact of impairment on the outcome of the competition [5], thus emphasizing factors like level of fitness, talent, motivation, and skill among players. From its first version, the classification has been revised to better achieve its goal, also thanks to a representation of the wheelchair rugby population (N = 302), which underlined in a survey of 2009 the need for some adjustments [6]. That was the case, for instance, of the increase of the maximum score relative to the trunk evaluation from 1.0 to 1.5 introduced in 2010 [7].

Although “the evidence-base for supporting elite wheelchair sport performance remains in its infancy when compared to able-bodied (AB) sport” [8], the interest in sport performance of Paralympic wheelchair athletes has begun to grow among scientists. In the early studies, it has been demonstrated that the peak of oxygen consumption and the anaerobic capacity were inversely correlated with the spinal lesion’s level [9,10,11,12,13,14]. However, this research involved only participants with spinal cord injuries which do not represent all wheelchair rugby players. Subsequently, the performance analysis has taken hold, and research on wheelchair rugby concentrated mostly on the propulsion technique related to sprint performance [15,16], the activity [2], and speed [17] profiles during competition and training. Only two studies investigated the sport-specific performance of wheelchair rugby players concerning their IWRF classification score [18,19]. Although a general correlation with the activity profiles of the players in terms of peak speed and ability to perform high-intensity activity was found [19], non-significant differences were detected among adjacent classified groups (Group I, 0.5-point players; Group II, 1 and 1.5-point players; Group III, 2 and 2.5-point players; and Group IV, 3 and 3.5-point players) regarding aerobic, anaerobic and sport-specific skill performance, separately [18]. Therefore, the authors suggested a continued examination of the IWRF classification system—emphasizing on-court performance and using alternate measures [18].

Since in wheelchair rugby, both aerobic and anaerobic capacities are required, due to the nature of the sport itself [18], the main limitation of previous investigations which deepened the relationship between sport-performance and IWRF classification was to consider a limited number of tests and to correlate them singularly with the IWRF classification. Therefore, in the present work, we considered (at the same time) a high number of aerobic and anaerobic tests considering the wheelchair rugby sport-specific requirements [2]. Our aim was twofold: (i) To investigate aerobic and anaerobic personalized tests in Paralympic athletes and their correlation with the IWRF classification; (ii) to investigate the overall level of sport-performance among adjacent classified groups of players. Our hypothesis was to find a moderate to a strong correlation between aerobic and anaerobic performance parameters and the IWRF classification points.

## 2. Materials and Methods

### 2.1. Participants

Sixteen male elite players of the Italian wheelchair rugby team were enrolled for the study (age 26 ± 6 yrs, body mass 72.5 ± 16.2 kg). Ten were tetraplegic (five C5/6; four C6/7; one T6/7), two affected by cerebral palsy, one by polio, one by transverse myelitis, one by Brown Sequard syndrome, and one by a rare syndrome which comprises a group of multiple congenital anomaly disorders. According to the IWRF classification, the group included two 0.5-point players, three 1.0-point players, three 1.5-point players, two 2.0-point players, three 2.5-point players, and three 3.5-point players. All players verbally reported a similar training level in their local teams, which was quantified in two weekly sessions of two hours each. A detailed description of the experimental procedures was given to each player, which provided written consent prior to testing. The study was approved by the ethical committee of the Department of Biomedical Sciences, University of Padova (HEC-DSB 02/16).

### 2.2. Study Overview

**Figure 1:**
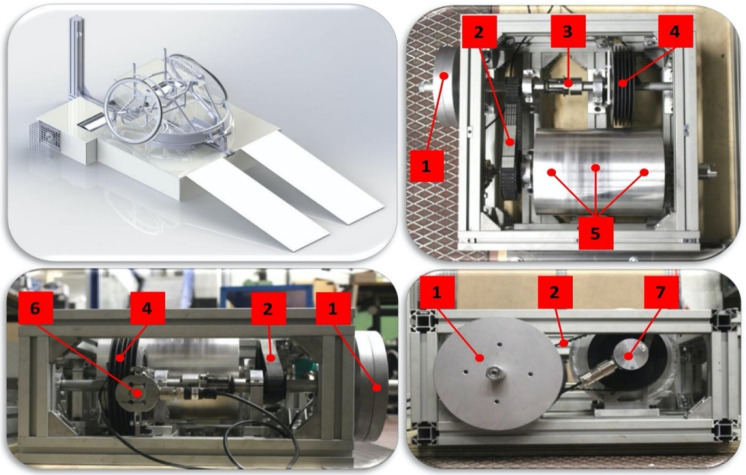
Non-motorized ergometer employed in the tests. Numbers are referred to: Inertial disks (**1**); transmission belt (**2**); mechanical coupling (**3**); electromechanical brake (**4**); drum (**5**); load cell (**6**); encoder (**7**).

Each player performed an incremental test to exhaustion, a Wingate 30s all-out sprint, and a sport-specific isometric test. The three tests were all performed on a non-motorized ergometer developed by the Department of Industrial Engineering of the University of Padova for wheelchair users (Figure 1). Briefly, the bench is composed of two independent drums connected by a shaft and can simulate the equivalent linear inertia of the wheelchair-athlete system detected in the court. Two electromechanical brakes restrained by two load cells are employed to increment the load. The sampling frequency to collect the instantaneous distance covered, linear speed and acceleration, braking torque, and power is set at 100 Hz. The accuracy and the reliability of the ergometer were measured, asking 10 wheelchair rugby players to perform three sprints of 20 m on-court, and the day after, three sprints of 20 m on the non-motorized ergometer. Besides the tests on the non-motorized ergometer, players performed sprints and one shuttle test on-court, the time of those was collected electronically through two photocells (Microgate, Bolzano, Italy).

**Figure 2:**
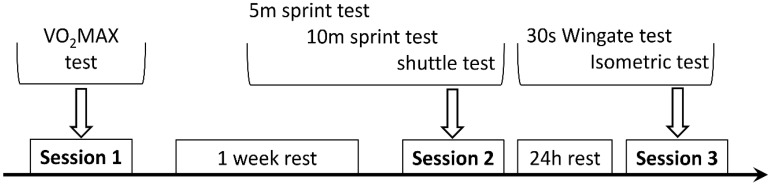
Schematic representation of the experimental protocol.

Tests were performed in three separate sessions (Figure 2): In the first, players carried out the incremental test to exhaustion. In the second, after one week, they performed the 5-meter and the 10-meter sprints followed by the shuttle test. In the third session, they performed the Wingate 30s all-out test, and after 5 min, the sport-specific isometric test. All players were verbally encouraged by researchers and coaches to do their best during the whole execution of the tests.

### 2.3. Incremental Test to Exhaustion

Ventilation (VE) and oxygen consumption (VO_2_) were measured breath-by-breath with open-circuit spirometry (Vmax, SensorMedics, Yorba Linda, CA, USA) during a continuous incremental test to voluntary exhaustion. More in detail, players had to maintain the same speed (5 km/h), while every 90 s, the resistance of the ergometer increased (5 watts for classification points ranging from 0.5 to 1.5 and 10 watts for classification points ranging from 2 to 3.5 [20]). The difference in the amount of increment was chosen to avoid too large power increments for the low pointers and an excessive duration of the test for the high pointers [21]. Since the non-motorized ergometer was able to simulate the equivalent linear inertia of the wheelchair-athlete system detected in the court, the power output of the first 90 s was for each player the resistance, while wheeling at 5 km/h on a flat path. After the application of a moving average filter, the peak of the oxygen consumption (VO_2_ peak) was defined as the highest value recorded during the incremental test and was expressed in milliliters per minute per kilogram of body mass.

### 2.4. Wingate All-Out Sprint Test

The all-out sprint test was based on the Wingate anaerobic test proposed elsewhere [21]. It consisted of a 30-second all-out effort on the non-motorized ergometer. The level of resistance initially set by the operator corresponded to the highest level reached during the previous maximal test to exhaustion. Then each player was asked to start wheeling at his maximum speed for 5 s to check that the peak velocity stayed below the 3 m·s^−1^ to prevent coordination problems [21]. If the peak velocity was higher than 3 m·s^−1^ the braking load was adjusted, and the player repeated the 5-second sprint to check the proper maximum speed [14]. At the end of this procedure, after a 5-minute rest, the player performed the Wingate 30-second all-out test. Among the parameters calculated, the highest mean power over successive 5-second intervals (P5) and the mean power over 30 s (P30) have already been proposed by Van der Scheer et al. [14]. In addition to these, we calculated the mean power of the last 5-second interval (FP5), the absolute peak power output in the first 5 s (APPO), the minimum peak power output in the last 5 s (MPPO), the integral mean of the power signal over thrusts, including APPO and MPPO (MAPPO and MMPPO, respectively).

### 2.5. Isometric Test on the Drums

The sport-specific isometric test has already been employed to study muscular strength in wheelchair users [21]. It quantifies the pushing strength derived from the synergic activity of the muscles involved in the pushing gesture. The drums of the non-motorized ergometer were blocked, and each player pushed as hard as he could for 5 s with hands on the upper part of the wheels. The force considered for the analysis was the sum of those collected by the two load cells for the right and the left wheels, respectively. Three trials separated by 2-minute rest were performed, and the best one was chosen for the analysis.

### 2.6. On-Court Sprint Tests

We implemented a set of tests that considered the on-court requirements of wheelchair rugby [2,3]. Players performed three linear sprints of 5 m followed by three linear sprints of 10 m, which reflected an acceleration from standstill. Then, they performed a shuttle test, which mimicked the on-court braking/acceleration actions. The shuttle test consisted of a 4-meter forward sprint followed by a 2-meter backward sprint to be repeated for a total of 28 m at the maximum speed (20 m forward and 8 m backward). Recovery of 2 min occurred at the end of each trial. The time of the best trial of both the 5 and the 10-meter sprints together with the time of the shuttle test was considered for the analysis.

### 2.7. Statistical Analysis

Spearman’s rank correlation coefficient was determined between the performance score of each parameter and the corresponding IWRF classification point. The IWRF classification point was also correlated with the sum of the Z scores relative to each test. An alpha level of *p* < 0.05 was set as a criterion for statistical significance. Correlation coefficients, which were ≤0.35, were considered weak, 0.36 to 0.67 moderate correlations, and 0.68 to 1.0 strong correlations. The analysis was performed with the software package GraphPad Prism version 4.00 for Windows (GraphPad Software, San Diego, CA, USA). Moreover, players were divided into three groups according to their IWRF classification points (Group 1: [0–1]; Group 2: [1–2]; Group 3: [2–3.5]) to carry out a multivariate permutation-based ranking analysis [22,23]. This analysis detects the differences in the overall level of performance among the three groups considering the scores of each test.

## 3. Results

The descriptive results of all tests are reported in Table 1. Data are presented as means and standard deviations. In the preliminary study, the non-motorized ergometer showed a good accuracy (the mean time measured on the ergometer was ≃7% higher than the mean time measured on-court) and reliability (ICC = 0.94).

### 3.1. Incremental Test to Exhaustion

In the incremental test to voluntary exhaustion the IWRF classification correlated with the VO_2_ peak (r = 0.58; *p* = 0.0171; 95% C.I. = 0.11 to 0.84), the VE peak (r = 0.62; *p* = 0.0106; 95% C.I. = 0.16 to 0.86) and the peak power output (r = 0.79; *p* = 0.0003; 95% C.I. = 0.47 to 0.93). The correlation of the peak power output normalized to the athletes’ body mass did not result statistically significant (r = 0.48; *p* = 0.0584; 95% C.I. = −0.03 to 0.80).

### 3.2. Wingate All-Out Sprint Test and Isometric Test

In the Wingate 30s all-out test the IWRF classification correlated with P5 (r = 0.81; *p* = 0.0001; 95% C.I. = 0.53 to 0.94), P5 normalized to athletes’ body mass (r = 0.61; *p* = 0.0127; 95% C.I. = 0.14 to 0.85), P30 (r = 0.81; *p* = 0.0002; 95% C.I. = 0.51 to 0.93), P30 normalized to athletes’ body mass (r = 0.61; *p* = 0.0114; 95% C.I. = 0.15 to 0.86), FP5 (r = 0.81; *p* = 0.0001; 95% C.I. = 0.52 to 0.94), FP5 normalized to athletes’ body mass (r = 0.63; *p* = 0.0086; 95% C.I. = 0.18 to 0.86), APPO (r = 0.88; *p* = 0.0001; 95% C.I. = 0.66 to 0.96), APPO normalized to athletes’ body mass (r = 0.73; *p* = 0.0014; 95% C.I. = 0.35 to 0.90), MPPO (r = 0.71; *p* = 0.0020; 95% C.I. = 0.32 to 0.90), MPPO normalized to athletes’ body mass (r = 0.61; *p* = 0.0124; 95% C.I. = 0.15 to 0.85), MAPPO (r = 0.91; *p* = 0.0001; 95% C.I. = 0.75 to 0.97), MAPPO normalized to athletes’ body mass (r = 0.68; *p* = 0.0034; 95% C.I. = 0.27 to 0.89), MMPPO (r = 0.81; *p* = 0.0001; 95% C.I. = 0.52 to 0.94), MMPPO normalized to athletes’ body mass (r = 0.61; *p* = 0.0127; 95% C.I. = 0.14 to 0.85). The peak force recorded during the sport specific isometric test correlated with the IWRF classification (r = 0.74; *p* = 0.0011; 95% C.I. = 0.37 to 0.91), but not when values were normalized to the athletes’ body weight (r = 0.33; *p* = 0.2180; 95% C.I. = −0.22 to 0.72).

### 3.3. On-Court Sprint Tests

The on-court sprint tests showed a high reliability with an ICC ranging from 0.92 (5-meter sprint test) to 0.99 (10-meter sprint test). A statistically significant correlation was detected between the IWRF classification point and the 5 m sprint (r = −0.67; *p* = 0.0049; 95% C.I. = −0.88 to −0.24), the 10 m sprint (r = −0.74; *p* = 0.0010; 95% C.I. = −0.91 to −0.38) and the shuttle sprint (r = −0.55; *p* = 0.0260; 95% C.I. = −0.83 to −0.063).

### 3.4. Z Score and Multivariate Permutation-Based Ranking Analysis

**Figure 3:**
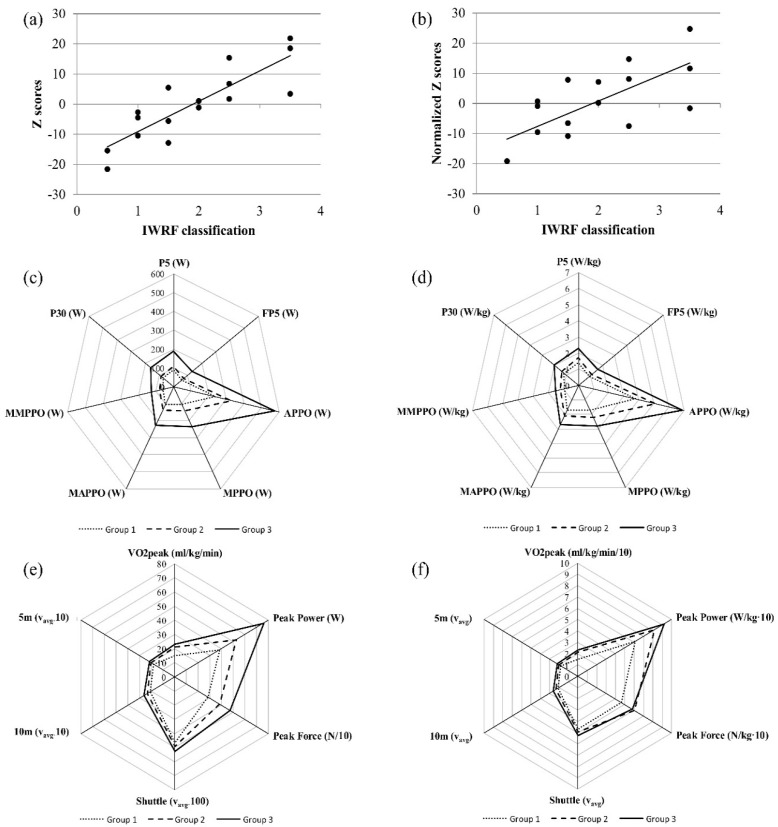
Z scores and permutation-based ranking analysis results. Absolute values are presented on the left (**a**,**c**,**e**); body mass normalized values are presented on the right (**b**,**d**,**f**). (VO_2_ peak, peak of the oxygen consumption; P5, highest mean power over a successive 5-second interval; P30, mean power over 30 s; FP5, mean power of the last 5-second interval; APPO, absolute peak power output in the first 5 s; MPPO, minimum peak power output in the last 5 s; MAPPO, integral mean of the power signal over thrusts including APPO; MMPPO, integral mean of the power signal over thrusts including MPPO).

The overall Z scores correlated with the IWRF classification points (r = 0.87; *p* = 0.0001; 95% C.I. = 0.65 to 0.96) also when Z scores were normalized to athletes’ body mass (r = 0.67; *p* = 0.0046; 95% C.I. = 0.25 to 0.88) (Figure 3a,b).

The multivariate permutation-based ranking analysis showed an overall better performance of the group 3 (IWRF classification points between [2–3.5]) with respect to group 1 (IWRF classification points between [0–1]; *p* = 0.0075) and group 2 (IWRF classification points between [1–2]; *p* = 0.0195). No statistically significant differences were detected between group 1 and group 2 (Figure 3c,e; *p* = 0.0950). Moreover, the multivariate permutation-based ranking analysis showed no statistically significant differences among the three groups when considering the results normalized to athletes’ body mass (Figure 3d,f).

## 4. Discussion

The present study aimed to investigate aerobic and anaerobic personalized tests in Paralympic wheelchair rugby players and to correlate them with the IWRF classification. The novelty of the study regards the consideration of a high number of aerobic and anaerobic parameters, both singularly and grouped, related to the wheelchair rugby sport-specific requirements. The main results of the present study showed statistically significant moderate to strong correlations between the performance parameters collected in the personalized tests and the IWRF classification. Only the normalized peak power output of the incremental test to exhaustion and the normalized peak force of the isometric strength test showed weak correlations with the IWRF classification. Moreover, correlations detected for the aerobic performance were substantially weaker than those referred to the anaerobic performance. These findings are comparable with the results of Morgulec-Adamowicz et al. where aerobic performance resulted in less sensitivity for identifying differences between grouped IWRF classes [18]. The strong correlations referred to the Wingate test, the isometric test, and the sprint tests could be explained by the specificity of the wheelchair rugby, where short and intense exercises have been detected [24]. Therefore, although it has been pointed out the relevance of the aerobic system [20], our results would seem to suggest, including in the wheelchair rugby performance assessment a Wingate test, an isometric sport-specific test, and a 10-meter sprint rather than an incremental test to exhaustion and a shuttle test.

However, it must be taken into account that wheelchair rugby proficiency depends on multiple activities as initial acceleration, 10-meter sprint, and hitting [25]. Thus, the single correlation of each test with the IWRF classification could be reasonably considered incomplete since it accounts for only the sole aspects of the performance. In this way, the overall ability of the player is difficult to predict. To overcome this limitation, we introduced the Z standard scores and the multivariate permutation-based ranking analysis. Indeed, Z standard scores revealed to be a good representation of the overall level of performance of each player and showed a strong positive correlation with the IWRF classification. Conversely, the multivariate permutation-based ranking analysis underlined an overall better performance only of the group 3 with respect to group 1 and group 2, highlighting a similar level of performance between low pointers of group 1 and mid pointers of group 2. Therefore, our findings would seem to suggest excluding from the overall assessment the tests with the weakest correlations (i.e., the aerobic test, the shuttle test, and the 5-meter sprint) and to study the introduction of new objective aspects of the performance (e.g., maneuverability) which should consider not only the sport-specific impact of muscle weakness (i.e., strength impairment), but also the ability to produce strength in the effective direction (i.e., coordination impairment). The aim would be to better distinguish the overall level of performance among players, above all between low pointers and mid pointers. This approach could be suiTable for other Paralympic disciplines, where only physiological performance determinants are usually investigated [26].

Finally, the correlation values when performance was normalized to the players’ body mass deserve attention. Indeed, we found a decrease in the strength of Spearman’s correlations. This was evident for the peak power output relative to the incremental test to exhaustion, and above all, for the sport-specific isometric test. Moreover, the multivariate permutation-based ranking analysis with data normalized to the players’ body mass showed no statistically significant differences among the three groups. These findings raise for the first time the question if the body mass of the wheelchair players, which has an important role during blocking, should be addressed in the IWRF classification.

In the end, some limitations have to be acknowledged. First, we only studied the players of the Italian national team, and thus, our results might not necessarily be representative of all the other wheelchair rugby national teams. This could be due to the IWRF world ranking position of the Italian team (29° out of 35°) and to the absence of 3-point players. Second, the reduced number of the Italian national team players did not allow us to create more than three groups to perform the multivariate permutation-based ranking analysis. Clearly, having a higher sample size, best would have been to increase the number of groups to better distinguish the players’ characteristics within each group. Lastly, our study compared only physiological parameters to the IWRF classification score. Indeed, these parameters alone are not able to explain the complex factors underlying the IWRF classification.

## 5. Conclusions

Our study underlined moderate to strong correlations of aerobic and anaerobic performance parameters with the IWRF classification. Our suggestion is to consider only the anaerobic tests, due to their strong correlations with IWRF classification. However, wheelchair rugby proficiency depends on multiple activities [24,25], and on this point, multivariate permutation-based ranking analysis highlights a similar overall performance between low pointers of group 1 and mid pointers of group 2. Therefore, these results seem to point out the need to investigate new objective aspects of the performance, and to combine them with the physical assessment and qualitative observation during training and matches. This multidisciplinary approach could stimulate a more personalized approach to the performance assessment of wheelchair rugby players. Moreover, it could be useful to improve the adequacy of the IWRF classification in reducing the impact of impairment on the outcome of the competitions.

## Figures and Tables

**Table 1:** Descriptive results of the aerobic and anaerobic tests of the Italian wheelchair rugby team (VO_2_ peak, peak of the oxygen consumption; VE, ventilation; P5, highest mean power over a successive 5-second interval; P30, mean power over 30 s; FP5, mean power of the last 5-second interval; APPO, absolute peak power output in the first 5 s; MPPO, minimum peak power output in the last 5 s; MAPPO, integral mean of the power signal over thrusts including APPO; MMPPO, integral mean of the power signal over thrusts including MPPO).

	Descriptive Statistics		
	Mean ± SD	95% CI	
**Incremental test to exhaustion**			
VO_2_ peak (ml·min^−1^·kg^−1^)	19.83 ± 6.54	16.63	23.03
VE peak (l·min^−1^)	52.94 ± 20.24	43.04	62.84
Peak Power (W)	56.83 ± 23.18	45.83	67.83
Peak Power (W/kg)	0.79 ± 0.30	0.64	0.94
**Wingate 30 s all-out test**			
P5 (W)	132.31 ± 56.10	105.31	159.31
P5 (W/kg)	1.81 ± 0.64	1.5	2.12
P30 (W)	113.17 ± 51.15	88.17	138.17
P30 (W/kg)	1.55 ± 0.59	1.26	1.84
FP5 (W)	90.23 ± 43.04	69.23	111.23
FP5 (W/kg)	1.24 ± 0.50	1	1.48
APPO (W)	393.16 ± 180.49	305.16	481.16
APPO (W/kg)	5.36 ± 2.05	4.36	6.36
MPPO (W)	165.23 ± 79.93	126.23	204.23
MPPO (W/kg)	2.27 ± 0.94	1.81	2.73
MAPPO (W)	161.09 ± 70.31	127.09	195.09
MAPPO (W/kg)	2.21 ± 0.78	1.83	2.59
MMPPO (W)	90.00 ± 38.50	71	109
MMPPO (W/kg)	1.23 ± 0.42	1.02	1.44
**Isometric test**			
Peak force (N)	385.16 ± 104.67	334.16	436.16
Peak force (N/BW)	0.55 ± 0.14	0.48	0.62
**On-Court tests**			
5-meter sprint (s)	2.50 ± 0.33	2.34	2.66
10-meter sprint (s)	4.26 ± 0.65	3.94	4.58
Shuttle (s)	24.19 ± 2.46	22.99	25.39
